# Presence of *Cryptosporidium* spp and other enteroparasites with pathogenic potential in hemodialysis patients: an open controlled study

**DOI:** 10.1590/2175-8239-JBN-2024-0015en

**Published:** 2024-12-20

**Authors:** Yara Leite Adami, Nycole Abreu Gama, Flavia de Souza Cunha, Regina Helena Saramago Peralta, Jocemir Ronaldo Lugon

**Affiliations:** 1 Universidade Federal Fluminense, Departmento de Patologia, Faculdade de Medicina, Niterói, RJ, Brazil.; 2 Universidade Federal Fluminense, Programa de Pós-Graduação em Ciências Médicas, Faculdade de Medicina, Niterói, RJ, Brazil.; 3 Universidade Federal Fluminense, Faculdade de Medicina, Departamento de Medicina Clínica, Niterói, RJ, Brazil.

**Keywords:** Renal Insufficiency, Chronic, Renal Dialysis, Parasites, Cryptosporidium, Laboratory Diagnosis

## Abstract

**Introduction:**

The World Health Organization (WHO) points out that infection by enteroparasites can affect ~3.5 billion people around the world. Hemodialysis (HD) patients may be more susceptible to infections by opportunistic pathogens due to impaired immune function. We evaluated enteroparasite infection in a sample of HD-patients from two dialysis centers and in a control group.

**Methods:**

Fecal samples were processed using the Hoffmann-Pons-Janner, Ritchie, Willis, and Rugai techniques. Patients with kidney failure from two dialysis centers undergoing HD for more than 3 months were included. The control group consisted of relatives of the patients without overt CKD. The TaqMan PCR and multiplex real-time PCR were carried out for detection of *Cryptosporidium* spp. and *C. parvum* and to differentiate the *Entamoeba (E.) histolytica/E. dispar* complex, respectively

**Results:**

A total of 97 HD patients and 42 controls were enrolled in the study. Fifty (51.5%) fecal samples from the HD group were positive for enteroparasites, as were 26 (61.9%) from the control group (P = 0.260). *S. stercoralis* was the single helminth detected and was only present in HD-patients. Coproscopy detected seven positive samples for the *E. histolytica/E. dispar* complex, three from HD patients and four from controls: by PCR, all samples were positive for the non-pathogenic *E. dispar*. Safranin-stained fecal smear slides were all negative for *Cryptosporidium* spp. However, by PCR, amplification for *Crypstosporidium* spp. was seen in six samples, all from the HD patients. Two of the species were classified as *C. hominis* by PCR-RFLP

**Conclusions:**

Enteroparasite infection as detected by traditional techniques were not more prevalent in HD patients, but *S. stercoralis* was only found in these patients. It is noteworthy that *Cryptosporidium spp*. infection, also affecting only HD patients, could only be detected by molecular biology techniques.

## Introduction

It is estimated that chronic kidney disease (CKD) affects over 10% of the general population corresponding to more than 800 million people with some degree of renal dysfunction worldwide^1^. Studies on the prevalence of individuals on renal replacement therapy in Brazil demonstrate that there were 758 patients per million population (pmp) on dialysis in 2022^2^. Kidney failure patients on hemodialysis (HD) may be more susceptible to infections by opportunistic pathogens due to impaired immune function^3^.

Estimates from the World Health Organization (WHO) reports point out that infections by intestinal parasites can affect around 3.5 billion people around the world, particularly in developing countries^4^. *Cryptosporidium* spp is a zoonotic protozoan from Apicomplexa phylum considered pathogenic and a major cause of diarrheal disease in immunosuppressed individuals. It can be found in the gastrointestinal tract of several hosts and is one of the most prevalent waterborne parasites worldwide^5^. In immunocompetent individuals, the infection is limited and tend to self-resolve. However, in immunosuppressed ones, it might be life-threatening. In patients with AIDS, cancer, or undergoing HD, for instance, *Cryptosporidium* spp infections can cause acute diarrhea, which is associated with substantial morbidity and mortality^6^.

Recent reports indicate that the prevalence rate of the parasite among kidney failure patients is high, especially in developing countries^7,8^. In Brazil, for instance, several studies have reported protozoan infections among these patients, with parasites such as *Blastocystis* spp, *Endolimax nana*, *Entamoeba coli*, *E*. *histolytica*/*E. dispar complex*, *Giardia intestinalis* and *Strongyloides stercoralis* found in their fecal samples^9,10^. HD patients may present with a wide spectrum of gastrointestinal symptoms for different reasons, making their correlation with enteroparasite infections difficult^11,12^. In this scenario, an active search for parasitic infections could allow adequate treatment leading to improved quality of life. In the present study, we provide recent data on the prevalence of enteroparasites in HD patients compared with a control group without kidney failure in two counties of the metropolitan region of the city of Rio de Janeiro, Brazil.

## Methods

A cross-sectional survey with a convenience sample was carried out from March to November 2019. Patients with kidney failure undergoing HD for more than 3 months from two dialysis centers located in the cities of Niteroi (Center 1) and Itaborai (Center 2), without gender or age restriction, were included. The control group consisted of relatives of the patients without overt CKD who lived in the same residence and were therefore subjected to the same possible risk factors for enteroparasite infection.

The study was approved by the Ethics Committee of the Medical School of Universidade Federal Fluminense, Niteroi, Rio de Janeiro, Brazil, Protocol number 1.147.848. An informed consent was obtained from all individuals enrolled in the study.

All participants completed a standardized clinical and epidemiological questionnaire addressing symptoms related to intestinal parasite infections and social and demographic characteristics. They were instructed to collect fecal samples using universal collectors and received two flasks: one with 10% formalin to collect three samples on alternate days for a maximum period of ten days, and a dry one, for a single fresh sample. The samples were transported in refrigerated boxes to the laboratory of parasitology of the university hospital to undergo coproparasitological studies.

### Stool Processing and Examination

Fecal samples were processed through the techniques of Hoffman, Pons and Janner (1934)^13^, Ritchie (1948)^14^, Willis (1921)^15^, and Rugai (1954)^16^. Duplicate slides of each sample were prepared, and readings were made separately by two operators. The fresh samples were used to perform direct examination and the Rugai technique, and aliquots were cryopreserved in order to carry out molecular tests to detect infections by *Cryptosporidium* spp through real time PCR. For microscopic detection of *Cryptosporidium* spp. oocyst, the safranin/methylene blue staining technique was employed^17^. For coproscopic examination, the sediments were observed under 100, 400, and 1000× magnification using an optical microscope (Nikon Eclipse E 200^®^).

DNA extraction: Total genomic DNA was extracted from 500 µL of 127 fresh stool samples using the FastDNA™ Spin Kit for Feces (MP Biomedicals, USA) according to the manufacturer’s protocol. Samples were disrupted in an FP120 cell disruptor (MP Biomedicals) at a speed of 5.5 m/s for 10 s. DNA extracts were stored at –20ºC until further processing.

### TaqMan PCR Assays

The TaqMan PCR procedure combined a duplex reaction for the detection of *Cryptosporidium* spp. and *C. parvum* and a simple reaction for the detection of *C. hominis*, as described previously^18,19^. A total of 127 DNA samples were analyzed. The assays were performed with a 7500 Real-Time PCR System (Thermo Fisher Scientific Inc., USA). Each 20-μL duplex reaction contained 10 μL of 2X Platinum Quantitative PCR SuperMix-UDG (Invitrogen, Thermo Fisher Scientific Inc., USA), 100 nM of each probe (JVAP 18S and JVAGP2), 250 nM of each primer (JVAF, JVAR, JVAGF, and JVAGR), and 5 μL of DNA. For the *C. hominis* assay, each 20-μL reaction contained 10 μL of 2X Platinum Quantitative PCR SuperMix-UDG (Invitrogen, Thermo Fisher Scientific Inc., USA), 250 nM of each primer (JVAF, JVAR, JVAGF, and JVAGR), 5 mM MgCl^2^, twice the probe concentration used for the duplex assay (200 nM) and 5 μL of DNA. The Taq Man PCR cycling conditions consisted of denaturation at 95ºC for 2 min followed by 45 cycles of denaturation at 94ºC for 10 s, annealing at 55ºC for 30 s, and extension at 72ºC for 20 s. All assays included positive controls (*C. hominis* and *C. parvum*) and negative controls (DNA extracted from fecal samples negative for any parasites). To investigate the presence of inhibitory substances, negative samples were contaminated with Cryptosporidium DNA around 10fg and subsequently submitted to TaqMan PCR.

### Genotyping Analysis

Six DNA *Cryptosporidium* spp. positive samples were also determined by nested PCR amplifying an 825-bp fragment of the small-subunit 18S rDNA gene and RFLP analysis of the secondary PCR products, using the restriction enzymes SspI (Thermo Fisher Scientific, Inc. Waltham, Ma, USA) and VspI (Thermo Fisher Scientific). Primers (18S-1F and 18S SSU-R2 – 1325bp; 18SN-2F and 18S SSU-R4X – 819-825 bp) and amplification conditions used for PCR-RFLP were adopted from previous publications^20^. Cycle conditions were as follows: one cycle of 94ºC for 5 min, 35 cycles of a denaturation step at 94ºC for 30 s, an annealing step at 56ºC for 45 s, and an extension step at 72ºC for 90 s, with a final extension for 10 min at 72ºC.

Enzymatic-digested products obtained from the six samples were analyzed on a 2% agarose gel and visualized by ethidium bromide staining. Samples that contained *C. parvum* and *C. hominis* were further subtyped by DNA sequencing of the gp60 gene amplified by a nested PCR following the protocol described by Glaberman et al.^21^. Each sample was amplified at least three times by PCR. Primers AL3531 and AL3533 (840 bp) were used in the primary PCR and primers AL3532 and LX0029 (440 bp) in the secondary PCR. The gp60 and 18S rRNA products were purified according to the manufacturer’s instructions using a NucleoSpin^®^ Extract II kit (MACHEREY-NAGEL GmbH and Co. KG, Germany). Sequencing was carried out in both directions. The nucleotide sequences obtained in this study were aligned with reference sequences retrieved from GenBank and gp60 sequences. The resulting sequences were edited and aligned with the Bioedit Sequence Alignment Editor 7.0.5.3. and MEGA 4.1^22^.

### Multiplex Real-Time PCR for Differentiation of *E. hystolitica*/*E. dispar* Complex

Real-time PCR-Multiplex was performed in seven microscopic positive samples according to the protocol of Gomes et al.^23^. The target sequences were amplified using a pair of oligonucleotide primers specific for each species^24^: *E. dispar* - EDP1 (5’ATGGTGAGGTTGTAG CAGA GA 3”) and o EDP2 (5’ CGATATTGACCTAGTACT 3’), generating a product of 96 base pairs (pb). *E. histolytica* - EHP1 (5’ CGATTTTCCCAGTAGAAATTA 3’) and EHP2 (5’ CAAAATGGTCGTCTAGGC 3’), generating a product of 132 pb. This reaction has a total volume of 25 μL and contains 0.5 μL of a 5 μM solution of each primer (EHP1, EHP2, EDP1 e EDP2), 1.25 pmoles of RoxReferenceDye (Invitrogen, Thermo Fisher Scientific Inc. USA), 8 µL of deionized water, 12.5 µL of Platinum SYBR Green qPCRSuperMix-UDG (Invitrogen, Thermo Fisher Scientific Inc. USA), and 2 µL of the purified DNA solution. The amplification reaction was performed using the ABI 7500 System thermocycler (Applied Biosystems, Thermo Fisher Scientific Inc. USA) and under the following conditions: an initial step at 50°C for two minutes, one step at 95°C for 2 minutes; 35 cycles composed of the stages of 15 seconds at 95°C and 33 seconds at 55°C; and a final stage corresponding to the dissociation curve, consisting of 15 seconds at 95°C, followed by 1 minute at 60°C, 15 seconds at 95°C, and 15 seconds at 60°C. The visualization of the amplification was obtained in the program ABI 7500 System Software (Thermo Fisher Scientific Inc. USA). The analysis was carried out in seven microscopic positive samples.

Statistical Analysis

Data analysis was performed using the software GraphPad Prism version 8.0 for Windows (www.graphpad.com). The Mann-Whitney test was employed to compare independent groups and differences among three or more groups were analyzed through Friedman ANOVA complemented by the Tukey test. Categorical variables were expressed as prevalence rates with differences tested using the chi-squared test. Statistical significance was set at P values < 0.05.

## Results

A total of 139 participants were enrolled in the study, 97 with kidney failure on HD and 42 controls. The mean age of HD patients was 57 ± 12 years and of the controls, 50±16 years (P = 0.01). In Center 1, 42 patients were included with a mean age of 59 years, the majority being female (52%). The 55 patients in center 2 tended to be younger, with a mean age of 55 years (P = 0.08) and the majority was male (62%). Data regarding social and demographic characteristics of all participants were described in a previous report^10^.

### Traditional Coproparasitological Studies

Fifty out of 97 (51.5%) fecal samples from 97 HD patients and 26 samples (61.9%) from 42 controls were positive for enteroparasites (P = 0.260). As shown in Table 1, the total prevalence of infection by enteroparasites was 54.7%, and protozoan species were more frequently found than helminths. The protozoan *Blastocystis* spp. was the most prevalent parasite, being detected in 42.5% of the samples, followed by *E. nana* (17.9%). In addition, *Entamoeba histolytica/E. dispar* (5.1%), *S. stercoralis* larvae (2.2%), and *E. coli* cysts (0.7%) were also observed. The parasitic infection profile was similar between the study groups with the exception of *S. stercoralis*, which was only found in 3 HD patients. Also, the prevalence of enteroparasites was similar between centers, both in the group of patients and in the control group (p > 0.05).

**Table 1:** Intestinal parasites detected by coproscopy in samples from hemodialysis (HD) patients and controls

	All	HD patients	Controls	P
n	139	97	42	
*Blastocystis* spp.	59 (42.5)	40 (41.3)	19 (45.3)	0.66
*Endolimax nana*	25 (17.9)	16 (16.5)	9 (21.4)	0.49
*E. histolytica/E. díspar*	7 (5.1)	3 (3.1)	4 (9.5)	0.11
*Strongyloides stercoralis*	3 (2.2)	3 (3.1)	0	-
*Entamoeba coli*	2 (1.4)	1 (1.1)	1 (2.4)	0.56
Negatives	63 (45.3)	47 (48.5)	16 (38.1)	0.25

Note – Data are expressed as n (%); E. = Entamoeba; *P* value obtained by proportion test.

Infection by a single parasitic species (monoparasitism) was more often found than mixed infections (polyparasitism) in both groups with statistical significance in HD patients (74 vs. 26%, P = 0.01), but not in controls (76.9 vs. 23.1%, P = 0.06). Seven samples were positive for the *E. histolytica*/*E. dispar* complex (three from HD patients and four from controls). A total of 139 sediments were obtained through the Ritchie technique and used for preparing 278 slides stained with safranin-methylene blue in order to carry out screening for *Cryptosporidium* spp oocysts. All samples analyzed were negative for parasite oocysts by microscopy.

### Molecular Analysis Findings

Multiplex real-time PCR confirmed the coproscopy results for *Entamoeba*, detecting three positive samples from HD patients and four from controls in the same cases, with all samples amplifying for the non-pathogenic *E. dispar*.

**Figure 1:**
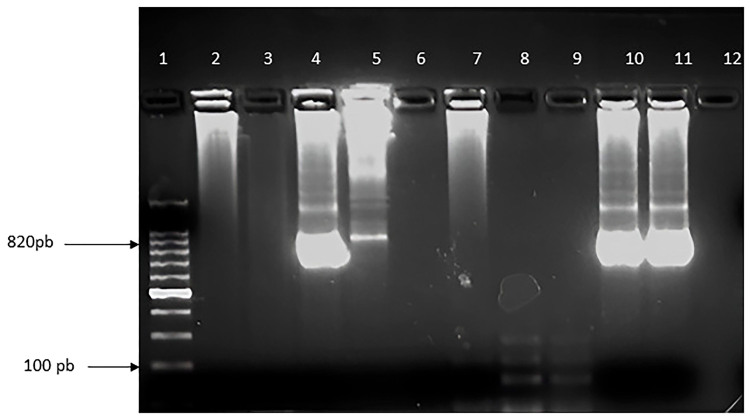
Amplification of a fragment of the 18S rRNA region from stool samples from participants with *Cryptosporidium* spp. infection by nested-PCR. Notes – Columns: 1. Molecular weight standard (Invitrogen, Thermofisher Scientific), 2. Sample P1, 3. Sample P19, 4. Sample P30, 5. Sample P32, 6. Sample P33, 7. Sample P39, 8. Blank, 9. Negative Control, 10. Positive Control *C. hominis*, 11. Positive Control *C. parvum*. Samples were observed in 2% agarose gel stained with ethidium bromide.

**Figure 2:**
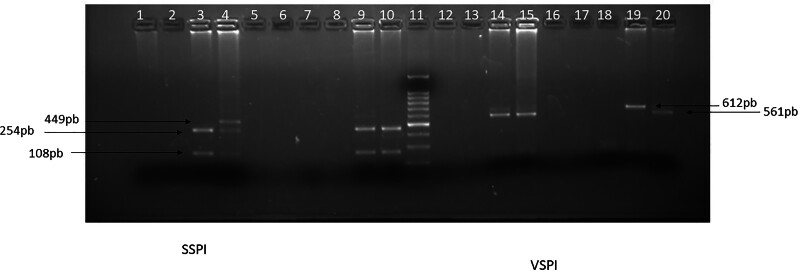
Digestion profile of the 18S rDNA fragment by the restriction enzymes VspI and SspI from the participants’ stool samples. Notes – Columns: 1. Sample P1, 2. Sample P19, 3. Sample P30, 4. Sample P32, 5. Sample P33, 6. Sample P39, 7. Blank, 8. Negative Control, 9. Positive Control *C. parvum*, 10. Positive Control *C. hominis*, 11. Molecular weight standard (Invitrogen, Thermofisher Scient.), 12. Sample P1, 13. Sample P19, 14. Sample P30, 15. Sample P32, 16. Sample P33, 17. Sample P39, 18. Negative Control, 19. Positive Control *C. parvum*, 20. Positive Control *C. hominis*. Samples were observed in 2% agarose gel stained with ethidium bromide.

Real time PCR detected the presence of *Cryptosporidium* spp. in 6 samples, all from HD patients. By this diagnostic method, despite the detection of the genus, it was not possible to differentiate the species of this parasite using probes to detect *C. hominis* and *C. parvum*. Samples considered positive for *Cryptosporidium* spp. through real time PCR were submitted to the nested PCR 18S rDNA. Observation by ultraviolet light revealed that two samples showed DNA amplification (Figure 1). Then, RFLP was performed and the interpretation of the agarose gel under ultraviolet light revealed the presence of samples classified as *C. hominis.* The two samples of *C. hominis* were subtyped with the nested PCR *gp60* gen and subtype IbA10G2 was determined (Figure 2). Positive diagnosis for *Cryptosporidium* spp. was not associated with symptoms in 5 patients (83.3%); one patient reported constipation.

## Discussion

Studies evaluating parasitism in HD patients show relevant rates of infection by enteroparasites, mainly caused by protozoa^12^, and most studies compared results of HD patients with those of subjects seen in outpatient clinics. A differential aspect of the present study is that the control group consisted of family members of the patients who lived in the same household and shared the same socio-environmental factors. This allowed a more reliable assessment of whether HD patients are more likely to acquire a parasitic infection or not.

As far as traditional coproscopic techniques are concerned, no significant differences were found regarding the prevalence of enteroparasitism between HD patients and controls in this study in both centers. The topic is controversial, with some authors reporting a higher prevalence of enteroparasitism in HD patients^9^, while others do not^25,26^. *Strongyloides stercoralis* was the only helminth detected in our study, and it is worth mentioning that its presence was restricted to samples from HD patients. Three patients had the parasite, and the diagnosis was promptly reported to the health care team. The frequently asymptomatic or nonspecific nature of active *Strongyloides* infection leads to underdiagnosis and puts immunosuppressed patients at increased risk of developing hyperinfection with dismal outcome^27^. There are some studies that report hyperinfection by *S. stercoralis* as a consequence of the use of immunosuppressive drugs in kidney transplantation^26,27^. Interestingly, among the previously cited studies, *S. stercoralis* has been described only in Brazilian studies and among individuals on HD, however, studies performed in Bolivia were also able to demonstrate that the parasite was prevalent among patients undergoing HD evaluated by serological and coproparasitological tests^28^.

Monoparasitism was more common than polyparasitism among individuals on dialysis patients (74%) and controls (76.9%). These results are in line with those reported by other authors^25,26^. The difference, however, (mono vs. polyparasitism) was significant only in the HD group (P = 0.01), while in the controls, a trend was observed (P=0.06), perhaps due to the smaller number of participants in this group.

Most of the patients seen at the clinics came from the public health system and had a low socioeconomic status. It is known that education is an important factor in preventing enteroparasitism. Access to treated water is also an important factor since horizontal contagion is often due to water contaminated with parasites, especially protozoa^29^.

Infections caused by protozoa were more frequent than those caused by helminths in both study groups, with a great diversity of protozoan organisms. The prevalence rate of infection in HD patients was similar to another Brazilian study^9^, but higher than that found in other studies carried out in Brazil^12^ and in other nations^25,26^.

The diagnosis of the *Entamoeba histolytica/E. dispar* complex by coproscopy was positive in 7 samples, 3 from patients and 4 from controls. Real time PCR confirmed these findings, showing similar sensitivity to direct parasite identification in our system. Although the detection of amoebic infections is equivalent compared to coproscopy, the molecular technique has the advantage to differentiate the species. It was observed that the samples considered to be *Entamoeba histolytica*/*E. dispar* by coproscopy were actually *E. dispar* by real time PCR. This differentiation is important, as the amoeba detected is considered non-pathogenic and rarely affects the health of the host, while *E. histolytica* can cause amebiasis with unpredictable course, which may include severe gastrointestinal symptoms and bloody dysentery^30^.

Fotedar et al.^31^ investigated the prevalence of amoeba cysts in symptomatic patients through microscopy and molecular diagnosis. Coproscopy revealed amoebic complexes in 2.9% of the samples and the molecular diagnosis revealed that the species *E. dispar* and *E. moshkovskii* were the most commonly found. In fact, current data indicates that infections caused by *E. dispar* are more common than those caused by *E. histolytica* in the world^31-33^. In this regard, Calegar et al.^34^ reported a prevalence of amoeba infection of 10.3%, and species differentiation through PCR was effective in 21 of the 23 samples tested, showing that the non-pathogenic *E. dispar* prevailed (57.1%). The differentiation of amoeba species is relevant, since a significant number of patients may be treated with antiparasitic drugs such as metronidazole without being infected with *Entamoeba histolytica* but rather with *E. dispar,* which is believed to be non-pathogenic^34^.

Laboratory diagnosis of cryptosporidiosis in stool samples can be made through microscopic examination of the stained slides, immunochromatography, ELISA, and molecular methods. Our results corroborate those of Abdel-Gawad et al.^35^ (2018), in which they compared the effectiveness of three different diagnostic methods (PCR, ELISA, and coproscopy) and considered PCR as the gold standard, with a 100% sensitivity and specificity. Ghallab et al. (2022) also expressed the opinion that the PCR should replace other detection methods in the near future, becoming the gold standard for the detection of *Cryptosporidium* spp^36^.

In the present study, 6 samples were positive for *Crypstosporidium* spp., all from HD patients, with 2 being classified as *C. hominis* by PCR-RFLP. The remaining 4 samples showing amplification for the specific genus probe may be compatible with species other than *C. hominis* and *C. parvum*. The non-detection of the related species may be linked to the fact that they are species of lower infectious power and the parasitic load is small, confirmed by the lack of signs and symptoms. The parasitic load was probably low, since only real time PCR was able to detect the presence of *Criptosporidium* spp genetic material. The two samples were characterized for species and subtype *C. hominis* IbA10G2. This subtype is described in the literature as cosmopolitan, more virulent, and with the genotype most likely to undergo recombination^37^. This subtype predominates in northern Europe and has been reported in outbreaks in the United Kingdom, generally associated with recreational waters^38^. However, the IbA10G2 subtype is cosmopolitan. In addition to Europe, it was found in approximately 50% of outbreaks associated with *C. hominis* in the USA, and in a study conducted in Peru, it was considered the most virulent subtype^5^. In Brazil, only one study has investigated the presence of IbA10G2 subtype and found positivity in patients from the same region as in the present study^19^. The determination of the subtypes helps us to better understand how the parasite behaves within the host, which is important to the development of new strategies for the prevention and treatment against cryptosporidiosis^39^.

In our view, the inclusion of molecular diagnostics in the laboratory routine is an attractive alternative to microscopic diagnostics, efficiently saving laboratory time. In addition, the technique does not require preservative fluids, such as formalin, which may be carcinogenic. It is a technique with very good sensitivity and specificity, which prevents false-negative results and allows differentiation of parasites described on the reports as “complex”, for instance, *E. histolytica* / *E. dispar* ^40^.

The study has limitations. The sample is relatively small and the control group, which depended on voluntary participation, was smaller than expected. The decision to include controls of the same household as HD patients and the use of advanced molecular biology techniques are the study’s strengths.

Our results indicate that HD patients did not have higher prevalence rates of enteroparasite infection. Protozoan species were frequently found in the coproparasitological tests from participants from both centers and in patients and controls, perhaps reinforcing the need for diagnosis and treatment of asymptomatic patients. Noteworthy, the detection of *S. stercoralis* and *Cryptosporidium* spp was restricted to HD patients. Our data highlight the need for implementation of unconventional diagnostic techniques that can act as a differential factor in the detection of enteroparasites, especially in specific groups, such as HD patients.
