# Supplementary material for: Presence of Cryptosporidium spp and other enteroparasites with pathogenic potential in hemodialysis patients: an open controlled study
Source: J Bras Nefrol. 2024 Dec 20;47(1):e20240015. doi: 10.1590/2175-8239-JBN-2024-0015en (PMC11755876; doi:10.1590/2175-8239-JBN-2024-0015en)
Supplement: Translation

# Translation: Presença de *Cryptosporidium* spp e outros enteroparasitos com potencial patogênico em pacientes em hemodiálise: um estudo aberto e controlado

**DOI:** 10.1590/2175-8239-JBN-2024-0015pt

**Authors:** Yara Leite Adami, Nycole Abreu Gama, Flavia de Souza Cunha, Regina Helena Saramago Peralta, Jocemir Ronaldo Lugon

**Affiliations:** 1 Universidade Federal Fluminense, Departmento de Patologia, Faculdade de Medicina, Niterói, RJ, Brasil.; 2 Universidade Federal Fluminense, Programa de Pós-Graduação em Ciências Médicas, Faculdade de Medicina, Niterói, RJ, Brasil.; 3 Universidade Federal Fluminense, Faculdade de Medicina, Departamento de Medicina Clínica, Niterói, RJ, Brasil.

**Keywords:** Insuficiência Renal Crônica, Diálise Renal, Parasitos, Cryptosporidium, Diagnóstico Laboratorial

## Abstract

**Introdução:**

A OMS aponta que infecções por enteroparasitos podem afetar ~3,5 bilhões de pessoas globalmente. Pacientes em hemodiálise (HD) podem ser mais suscetíveis a infecções por patógenos oportunistas devido à função imunológica prejudicada. Avaliamos a infecção por enteroparasitos em pacientes em HD de dois centros de diálise e um grupo controle

**Métodos:**

Amostras fecais foram processadas pelas técnicas de Hoffmann, Pons&Janner, Ritchie, Willis e Rugai. Incluímos pacientes com insuficiência renal, de dois centros de diálise, em HD por mais de três meses. O grupo controle consistiu em familiares dos pacientes sem DRC evidente. PCR TaqMan e PCR Multiplex em tempo real foram realizadas para detecção de *Cryptosporidium spp*. e *C. parvum* e para diferenciar o complexo *Entamoeba (E.) histolytica/E. dispar*, respectivamente

**Resultados:**

97 pacientes em HD e 42 controles foram incluídos no estudo. Cinquenta (51,5%) amostras fecais do grupo HD foram positivas para enteroparasitos, assim como 26 (61,9%) do grupo controle (P = 0,260). *S. stercoralis* foi o único helminto detectado, presente apenas nos pacientes em HD. A coproscopia detectou sete amostras positivas para o complexo *E. histolytica/E. dispar*, três de pacientes em HD e quatro controles: através da PCR, todas as amostras foram positivas para *E. dispar* não patogênica. As lâminas de esfregaço fecal coradas com safranina foram todas negativas para *Cryptosporidium* spp. Entretanto, através da PCR, observou-se amplificação para *Crypstosporidium* spp. em seis amostras, todas de pacientes em HD. Duas das espécies foram classificadas como *C. hominis* por PCR-RFLP

**Conclusões:**

A infecção por enteroparasitos, detectada por técnicas tradicionais, não foi mais prevalente em pacientes em HD, mas o *S. stercoralis* foi encontrado exclusivamente entre eles. Vale ressaltar que a infecção por *Cryptosporidium* spp., que também afetou somente pacientes em HD, pôde ser detectada somente por técnicas de biologia molecular.

## Introdução

Estima-se que a doença renal crônica (DRC) afete mais de 10% da população geral, o que corresponde a mais de 800 milhões de pessoas com algum grau de disfunção renal em todo o mundo^1^. Estudos sobre a prevalência de indivíduos em terapia renal substitutiva no Brasil demonstram que, em 2022, haviam 758 pacientes por milhão de habitantes (pmh) em diálise^2^. Pacientes com insuficiência renal em hemodiálise (HD) podem ser mais suscetíveis a infecções por patógenos oportunistas devido à função imunológica comprometida^3^.

Estimativas da Organização Mundial da Saúde (OMS) apontam que as infecções por parasitos intestinais podem afetar cerca de 3,5 bilhões de pessoas mundialmente, principalmente nos países em desenvolvimento^4^. O *Cryptosporidium* spp. é um protozoário zoonótico do filo Apicomplexa, considerado patogênico e uma das principais causas de doenças diarreicas em indivíduos imunossuprimidos. Pode ser encontrado no trato gastrointestinal de diversos hospedeiros e é um dos parasitos de veiculação hídrica mais prevalentes em todo o mundo^5^. Em indivíduos imunocompetentes, a infecção é limitada e tende a se resolver espontaneamente. No entanto, em indivíduos imunossuprimidos, pode ser fatal. Em pacientes com AIDS, câncer ou submetidos à HD, por exemplo, as infecções por *Cryptosporidium* spp. podem causar diarreia aguda, que está associada a morbidade e mortalidade substanciais^6^.

Relatos recentes indicam que a taxa de prevalência do parasito entre pacientes com insuficiência renal é elevada, especialmente em países em desenvolvimento^7,8^. No Brasil, por exemplo, diversos estudos relataram infecções por protozoários entre esses pacientes, com parasitos como *Blastocystis* spp., *Endolimax nana*, *Entamoeba coli*, complexo *E. histolytica/E. dispar*, *Giardia intestinalis* e *Strongyloides stercoralis* encontrados em suas amostras fecais^9,10^. Os pacientes em HD podem apresentar um amplo espectro de sintomas gastrointestinais por diferentes razões, dificultando sua correlação com infecções por enteroparasitos^11,12^. Nesse cenário, uma busca ativa por infecções parasitárias pode possibilitar o tratamento adequado, levando à melhora da qualidade de vida. No presente estudo, fornecemos dados recentes sobre a prevalência de enteroparasitos em pacientes em HD em comparação com um grupo controle sem insuficiência renal, em dois municípios da região metropolitana da cidade do Rio de Janeiro, Brasil.

## Métodos

Foi realizada uma pesquisa transversal com amostra de conveniência, de março a novembro de 2019. Foram incluídos pacientes com insuficiência renal em tratamento de HD por mais de 3 meses provenientes de dois centros de diálise localizados nas cidades de Niterói (Centro 1) e Itaborai (Centro 2), sem restrição de sexo ou idade. O grupo controle consistiu em familiares dos pacientes, sem DRC evidente, que residiam na mesma casa e, portanto, estavam expostos aos mesmos possíveis fatores de risco para infecção por enteroparasitos.

O estudo foi aprovado pelo Comitê de Ética da Faculdade de Medicina da Universidade Federal Fluminense, Niterói, Rio de Janeiro, Brasil, Protocolo número 1.147.848. Um termo de consentimento livre e esclarecido foi assinado por todos os indivíduos participantes do estudo.

Todos os participantes preencheram um questionário clínico e epidemiológico padronizado, abordando sintomas relacionados a infecções por parasitos intestinais, além de características sociais e demográficas. Eles foram instruídos a coletar amostras fecais utilizando coletores universais e receberam dois frascos: um com formalina a 10% para a coleta de três amostras em dias alternados, por um período máximo de dez dias, e um seco, para uma única amostra fresca. As amostras foram transportadas em caixas refrigeradas para o laboratório de parasitologia do hospital universitário, onde foram submetidas a estudos coproparasitológicos.

### Processamento e Exame das Fezes

As amostras fecais foram processadas por meio das técnicas de Hoffman, Pons e Janner (1934)^13^, Ritchie (1948)^14^, Willis (1921)^15^, e Rugai (1954)^16^. Foram preparadas lâminas duplicadas de cada amostra, e as leituras foram realizadas separadamente por dois operadores. As amostras frescas foram utilizadas para realizar o exame direto e a técnica de Rugai, e alíquotas foram criopreservadas com o objetivo de realizar testes moleculares para detectar infecções por *Cryptosporidium* spp., por meio da PCR em tempo real. Para a detecção microscópica de oocistos de *Cryptosporidium* spp., foi empregada a técnica de coloração pela safranina/azul de metileno^17^. Para o exame coproscópico, os sedimentos foram observados com aumentos de 100, 400 e 1.000X em microscópio óptico (Nikon Eclipse E 200^®^).

Extração de DNA: O DNA genômico total foi extraído de 500 µL de 127 amostras de fezes frescas utilizando o FastDNA™ Spin Kit for Feces (MP Biomedicals, EUA) de acordo com o protocolo do fabricante. As amostras foram rompidas em um disruptor de células FP120 (MP Biomedicals) a uma velocidade de 5,5 m/s por 10 s. Os extratos de DNA foram armazenados a –20ºC até o processamento subsequente.

### Ensaios de PCR TaqMan

O procedimento da PCR TaqMan combinou uma reação duplex para a detecção de *Cryptosporidium* spp. e *C. parvum* e uma reação simples para a detecção de *C. hominis*, conforme descrito anteriormente^18,19^. Foram analisadas 127 amostras de DNA. Os ensaios foram realizados com um sistema 7500 para PCR em tempo real (Thermo Fisher Scientific Inc., EUA). Cada reação duplex de 20 μL continha 10 μL de 2X Platinum Quantitative PCR SuperMix-UDG (Invitrogen, Thermo Fisher Scientific Inc., EUA), 100 nM de cada sonda (JVAP 18S e JVAGP2), 250 nM de cada primer (JVAF, JVAR, JVAGF e JVAGR) e 5 μL de DNA. Para o ensaio de *C. hominis*, cada reação de 20 μL continha 10 μL de 2X Platinum Quantitative PCR SuperMix-UDG (Invitrogen, Thermo Fisher Scientific Inc., EUA), 250 nM de cada primer (JVAF, JVAR, JVAGF e JVAGR), 5 mM de MgCl^2^, o dobro da concentração da sonda utilizada para o ensaio duplex (200 nM) e 5 μL de DNA. As condições de ciclagem do PCR Taq Man consistiram em desnaturação a 95ºC por 2 min, seguida de 45 ciclos de desnaturação a 94ºC por 10 s, anelamento a 55ºC por 30 s e extensão a 72ºC por 20 s. Todos os ensaios incluíram controles positivos (*C. hominis* e *C. parvum*) e controles negativos (DNA extraído de amostras fecais negativas para qualquer parasito). Para investigar a presença de substâncias inibitórias, as amostras negativas foram contaminadas com aproximadamente 10fg de DNA de *Cryptosporidium* e, posteriormente, submetidas à PCR TaqMan.

### Análise de Genotipagem

Seis amostras positivas de DNA de *Cryptosporidium* spp. também foram determinadas por *nested* PCR, amplificando um fragmento de 825 pb do gene rDNA da subunidade menor 18S e por análise de RFLP dos produtos secundários de PCR, utilizando as enzimas de restrição SspI (Thermo Fisher Scientific, Inc. Waltham, Ma, EUA) e VspI (Thermo Fisher Scientific). Os primers (18S-1F e 18S SSU-R2 - 1325 pb; 18SN-2F e 18S SSU-R4X - 819-825 pb) e as condições de amplificação usados para PCR-RFLP foram adotados de publicações anteriores^20^. As condições de ciclagem foram as seguintes: um ciclo de 94ºC por 5 min, 35 ciclos de uma etapa de desnaturação a 94ºC por 30 s, uma etapa de anelamento a 56ºC por 45 s e uma etapa de extensão a 72ºC por 90 s, com uma extensão final de 10 min a 72ºC.

Os produtos resultantes da digestão enzimática obtidos das seis amostras foram analisados em um gel de agarose a 2% e visualizados por coloração com brometo de etídio. As amostras que continham *C. parvum* e *C. hominis* foram posteriormente subtipadas por sequenciamento de DNA do gene gp60 amplificado por *nested* PCR, seguindo o protocolo descrito por Glaberman et al.^21^. Cada amostra foi amplificada pelo menos três vezes pela PCR. Os primers AL3531 e AL3533 (840 bp) foram utilizados na PCR primária e os primers AL3532 e LX0029 (440 bp) na PCR secundária. Os produtos do gp60 e 18S rRNA foram purificados de acordo com as instruções do fabricante usando um kit NucleoSpin^®^ Extract II (MACHEREY-NAGEL GmbH and Co. KG, Alemanha). O sequenciamento foi realizado em ambas as direções. As sequências de nucleotídeos obtidas neste estudo foram alinhadas com sequências de referência recuperadas do GenBank e sequências de gp60. As sequências resultantes foram editadas e alinhadas com o Bioedit Sequence Alignment Editor 7.0.5.3. e o MEGA 4.1^22^.

### PCR em Tempo Real Multiplex Para Diferenciação do Complexo *E. hystolitica/E. dispar*

A PCR em tempo real Multiplex foi realizada em sete amostras microscópicas positivas de acordo com o protocolo de Gomes et al.^23^. As sequências-alvo foram amplificadas utilizando um par de oligonucleotídeos primers específicos para cada espécie^24^: *E. dispar* - EDP1 (5’ATGGTGAGGTTGTAG CAGA GA 3”) e EDP2 (5’ CGATATTGACCTAGTACT 3’), gerando um produto de 96 pares de bases (pb). *E. histolytica* - EHP1 (5’ CGATTTTCCCAGTAGAAATTA 3’) e EHP2 (5’ CAAAATGGTCGTCTAGGC 3’), gerando um produto de 132 pb. Esta reação tem um volume total de 25 μL e contém 0,5 μL de uma solução de 5 μM de cada primer (EHP1, EHP2, EDP1 e EDP2), 1,25 pmoles de RoxReferenceDye (Invitrogen, Thermo Fisher Scientific Inc., EUA), 8 µL de água deionizada, 12,5 µL de Platinum SYBR Green qPCRSuperMix-UDG (Invitrogen, Thermo Fisher Scientific Inc., EUA) e 2 µL da solução de DNA purificado. A reação de amplificação foi realizada com o termociclador ABI 7500 System (Applied Biosystems, Thermo Fisher Scientific Inc., EUA) e sob as seguintes condições: uma etapa inicial a 50°C por dois minutos, uma etapa a 95°C por 2 minutos; 35 ciclos compostos pelas etapas de 15 segundos a 95°C e 33 segundos a 55°C; e uma etapa final correspondente à curva de dissociação, que consiste em 15 segundos a 95°C, seguidos de 1 minuto a 60°C, 15 segundos a 95°C e 15 segundos a 60°C. A visualização da amplificação foi obtida no programa ABI 7500 System Software (Thermo Fisher Scientific Inc., EUA). A análise foi realizada em sete amostras microscópicas positivas.

Análise Estatística

A análise dos dados foi realizada utilizando o software GraphPad Prism versão 8.0 para Windows (www.graphpad.com). O teste de Mann-Whitney foi empregado para comparar grupos independentes e as diferenças entre três ou mais grupos foram analisadas por meio do teste de Friedman ANOVA, complementado pelo teste de Tukey. As variáveis categóricas foram expressas como taxas de prevalência, com diferenças testadas usando o teste qui-quadrado. A significância estatística foi definida como valores de P < 0,05.

## Resultados

No total, 139 participantes foram incluídos no estudo, 97 com insuficiência renal em HD e 42 controles. A idade média dos pacientes em HD foi de 57 ± 12 anos e a dos controles, de 50 ± 16 anos (P = 0,01). No Centro 1, foram incluídos 42 pacientes com uma média de idade de 59 anos, sendo a maioria do sexo feminino (52%). Os 55 pacientes do Centro 2 eram, em geral, mais jovens, com uma média de idade de 55 anos (P = 0,08) e a maioria era do sexo masculino (62%). Os dados referentes às características sociais e demográficas de todos os participantes foram descritos em um relato anterior^10^.

### Estudos Coproparasitológicos Tradicionais

Cinquenta de 97 (51,5%) amostras fecais de 97 pacientes em HD e 26 amostras (61,9%) de 42 controles foram positivas para enteroparasitos (P = 0,260). Conforme mostra a Tabela 1, a prevalência total de infecção por enteroparasitos foi de 54,7%, e espécies de protozoários foram encontradas com maior frequência do que helmintos. O protozoário *Blastocystis* spp. foi o parasito mais prevalente, sendo detectado em 42,5% das amostras, seguido por *E. nana* (17,9%). Além disso, também foram observados *Entamoeba histolytica/E. dispar* (5,1%), larvas de *S. stercoralis* (2,2%) e cistos de *E. coli* (0,7%). O perfil de infecção parasitária foi semelhante entre os grupos do estudo, com exceção do *S. stercoralis*, que foi encontrado somente em 3 pacientes em HD. Além disso, a prevalência de enteroparasitos foi semelhante entre os centros, tanto no grupo de pacientes quanto no grupo controle (p > 0,05).

**Tabela 1:** Parasitos intestinais detectados por coproscopia em amostras de pacientes em hemodiálise (HD) e controles

	Todos	Pacientes em HD	Controles	P
n	139	97	42	
*Blastocystis* spp.	59 (42,5)	40 (41,3)	19 (45,3)	0,66
*Endolimax nana*	25 (17,9)	16 (16,5)	9 (21,4)	0,49
*E. histolytica/E. dispar*	7 (5,1)	3 (3,1)	4 (9,5)	0,11
*Strongyloides stercoralis*	3 (2,2)	3 (3,1)	0	-
*Entamoeba coli*	2 (1,4)	1 (1,1)	1 (2,4)	0,56
Negativos	63 (45,3)	47 (48,5)	16 (38,1)	0,25

Notas – Os dados são expressos como n (%); E. = *Entamoeba*; valor de P obtido pelo teste de proporção.

A infecção por uma única espécie parasitária (monoparasitismo) foi encontrada com mais frequência do que as infecções mistas (poliparasitismo) em ambos os grupos, com significância estatística em pacientes em HD (74 vs. 26%; P = 0,01), mas não em controles (76,9 vs. 23,1%; P = 0,06). Sete amostras foram positivas para o complexo *E. histolytica/E. dispar* (três de pacientes em HD e quatro do grupo controle).

Um total de 139 sedimentos foi obtido por meio da técnica de Ritchie e utilizado para a preparação de 278 lâminas coradas com safranina-azul de metileno, a fim de realizar triagem para oocistos de *Cryptosporidium* spp. Todas as amostras analisadas foram negativas para oocistos de parasitos por microscopia.

### Achados da Análise Molecular

A PCR Multiplex em tempo real confirmou os resultados da coproscopia para *Entamoeba*, detectando três amostras positivas obtidas de pacientes em HD e quatro de controles nos mesmos casos, com todas as amostras amplificando para *E. dispar* não patogênica.

A PCR em tempo real detectou a presença de *Cryptosporidium* spp. em 6 amostras, todas de pacientes em HD. Por este método diagnóstico, apesar da detecção do gênero, não foi possível diferenciar as espécies deste parasito usando sondas para detectar *C. hominis* e *C. parvum*. As amostras consideradas positivas para *Cryptosporidium* spp. por meio de PCR em tempo real foram submetidas ao *nested* PCR para 18S rDNA. A observação por luz ultravioleta revelou que duas amostras apresentaram amplificação de DNA (Figura 1). Em seguida, foi realizada a RFLP e a interpretação do gel de agarose sob luz ultravioleta revelou a presença de amostras classificadas como *C. hominis.* As duas amostras de *C. hominis* foram subtipadas com o *nested* PCR do gene gp60 e o subtipo IbA10G2 foi determinado (Figura 2). O diagnóstico positivo para *Cryptosporidium* spp. não foi associado a sintomas em 5 pacientes (83,3%); um paciente relatou constipação.

**Figura 1:** Amplificação de um fragmento da região 18S rRNA de amostras de fezes obtidas de participantes com infecção por *Cryptosporidium* spp. por nested-PCR. Notas – Colunas: 1. Padrão de peso molecular (Invitrogen, Thermofisher Scient.), 2. Amostra P1, 3. Amostra P19, 4. Amostra P30, 5. Amostra P32, 6. Amostra P33, 7. Amostra P39, 8. Branco, 9. Controle Negativo, 10. Controle Positivo *C. hominis*, 11. Controle Positivo *C. parvum*. Gel de agarose a 2% corado com brometo de etídio.

**Figura 2:** Perfil de digestão do fragmento 18S rDNA pelas enzimas de restrição VspI e SspI das amostras de fezes dos participantes. Notas – Colunas: 1. Amostra P1, 2. Amostra P19, 3. Amostra P30, 4. Amostra P32, 5. Amostra P33, 6. Amostra P39, 7. Branco, 8. Controle Negativo, 9. Controle Positivo *C. parvum*, 10. Controle Positivo *C. hominis*, 11. Padrão de peso molecular (Invitrogen, Thermofisher Scient.), 12. Amostra P1, 13. Amostra P19, 14. Amostra P30, 15. Amostra P32, 16. Amostra P33, 17. Amostra P39, 18. Controle Negativo, 19. Controle Positivo *C. parvum*, 20. Controle Positivo *C. hominis*. Gel de agarose a 2% corado com brometo de etídio.

## Discussão

Estudos que avaliaram o parasitismo em pacientes em HD mostram taxas relevantes de infecção por enteroparasitos, causadas principalmente por protozoários^12^, e a maioria dos estudos comparou os resultados de pacientes em HD com os de indivíduos atendidos em clínicas ambulatoriais. Um aspecto diferencial do presente estudo é que o grupo controle foi composto por membros da família dos pacientes que residiam na mesma casa e compartilhavam os mesmos fatores socioambientais. Isso possibilitou uma avaliação mais confiável sobre a probabilidade de os pacientes em HD adquirirem ou não uma infecção parasitária.

No que diz respeito às técnicas coproscópicas tradicionais, não foram encontradas diferenças significativas quanto à prevalência de enteroparasitismo entre pacientes em HD e controles neste estudo, em ambos os centros. O assunto é controverso, com alguns autores relatando uma maior prevalência de enteroparasitismo em pacientes em HD^9^, enquanto outros não afirmam o mesmo^25,26^. *Strongyloides stercoralis* foi o único helminto detectado em nosso estudo, e cabe mencionar que sua presença foi restrita a amostras de pacientes em HD. Três pacientes apresentaram o parasito e o diagnóstico foi prontamente comunicado à equipe de saúde. A natureza frequentemente assintomática ou inespecífica da infecção ativa por *Strongyloides* leva ao subdiagnóstico e coloca os pacientes imunossuprimidos em risco aumentado de desenvolver hiperinfecção com desfecho desfavorável^27^. Existem alguns estudos que relatam hiperinfecção por *S. stercoralis* como consequência do uso de medicamentos imunossupressores no transplante renal^26,27^. Curiosamente, entre os estudos citados anteriormente, *S. stercoralis* foi descrito somente em estudos brasileiros e entre indivíduos em HD. No entanto, estudos realizados na Bolívia também conseguiram demonstrar que o parasito foi prevalente entre pacientes em HD, avaliados por testes sorológicos e coproparasitológicos^28^.

O monoparasitismo foi mais comum que o poliparasitismo entre indivíduos em diálise (74%) e controles (76,9%). Esses resultados estão em conformidade com os relatados por outros autores^25,26^. A diferença, porém, (mono vs. poliparasitismo) foi significativa apenas no grupo HD (P = 0,01), enquanto nos controles observou-se uma tendência (P = 0,06), possivelmente devido ao menor número de participantes desse grupo.

A maioria dos pacientes atendidos nas clínicas era proveniente do sistema público de saúde e apresentava baixo nível socioeconômico. Sabe-se que a educação é um fator importante na prevenção do enteroparasitismo. O acesso à água tratada também é um fator fundamental, uma vez que o contágio horizontal geralmente se deve à água contaminada com parasitos, especialmente protozoários^29^.

As infecções causadas por protozoários foram mais frequentes do que as causadas por helmintos em ambos os grupos estudados, com uma grande diversidade de organismos. A taxa de prevalência de infecção em pacientes em HD foi semelhante à de outro estudo brasileiro^9^, mas superior àquela encontrada em outros estudos realizados no Brasil^12^ e em outras nações^25,26^.

O diagnóstico do complexo *Entamoeba histolytica*/*E. dispar* por coproscopia foi positivo em 7 amostras, 3 de pacientes e 4 de controles. A PCR em tempo real confirmou esses achados, mostrando sensibilidade semelhante à identificação direta do parasito em nosso sistema. Embora a detecção de infecções amebianas seja equivalente quando comparada à coproscopia, a técnica molecular tem a vantagem de diferenciar as espécies. Observou-se que as amostras consideradas como *E. histolytica*/*E. dispar* por coproscopia eram, na verdade, *E. dispar* através da PCR em tempo real. Essa diferenciação é importante, uma vez que a ameba detectada é considerada não patogênica e raramente afeta a saúde do hospedeiro, enquanto *E. histolytica* pode causar amebíase com curso imprevisível, que pode incluir sintomas gastrointestinais graves e disenteria sanguinolenta^30^.

Fotedar et al.^31^ investigaram a prevalência de cistos de ameba em pacientes sintomáticos por meio de microscopia e diagnóstico molecular. A coproscopia revelou complexos amebianos em 2,9% das amostras e o diagnóstico molecular revelou que as espécies *E. dispar* e *E. moshkovskii* foram as mais comumente encontradas. Na verdade, os dados atuais indicam que as infecções causadas por *E. dispar* são mais comuns do que as causadas por *E. histolytica* em todo o mundo^31-33^. Nesse sentido, Calegar et al.^34^ relataram uma prevalência de infecção por amebas de 10,3%, e a diferenciação de espécies pela PCR foi eficaz em 21 das 23 amostras testadas, mostrando que a forma não patogênica *E. dispar* prevaleceu (57,1%). A diferenciação das espécies de amebas é relevante, visto que um número significativo de pacientes pode ser tratado com medicamentos antiparasitários, como o metronidazol, sem estar infectado com *E. histolytica*, mas sim com *E. dispar*, que se acredita ser não patogênica^34^.

O diagnóstico laboratorial da criptosporidiose em amostras de fezes pode ser realizado por meio de exame microscópico das lâminas coradas, imunocromatografia, ELISA e métodos moleculares. Nossos resultados corroboram os de Abdel-Gawad et al.^35^ (2018), nos quais foram comparadas a eficácia de três métodos diagnósticos diferentes (PCR, ELISA e coproscopia) e a PCR foi considerada o padrão-ouro, com sensibilidade e especificidade de 100%. Ghallab et al. (2022) também expressaram a opinião de que a PCR deve substituir outros métodos de detecção em um futuro próximo, tornando-se o padrão-ouro para a detecção de *Cryptosporidium* spp.^36^.

No presente estudo, 6 amostras foram positivas para *Crypstosporidium* spp. todas de pacientes em HD, com 2 sendo classificadas como *C. hominis* por PCR-RFLP. As quatro amostras restantes que apresentaram amplificação para a sonda específica de gênero podem ser compatíveis com outras espécies além de *C. hominis* e *C. parvum*. A não detecção de espécies relacionadas pode estar ligada ao fato de serem espécies de menor poder infeccioso e a carga parasitária ser reduzida, o que é confirmado pela ausência de sinais e sintomas. A carga parasitária era provavelmente baixa, uma vez que somente a PCR em tempo real foi capaz de detectar a presença de material genético de *Criptosporidium* spp. As duas amostras foram caracterizadas para a espécie e o subtipo *C. hominis* IbA10G2. Esse subtipo é descrito na literatura como cosmopolita, mais virulento e com o genótipo com maior probabilidade de sofrer recombinação^37^. Esse subtipo predomina no norte da Europa e foi relatado em surtos no Reino Unido, geralmente associados a águas recreativas^38^. Entretanto, o subtipo IbA10G2 é cosmopolita. Além da Europa, foi encontrado em aproximadamente 50% dos surtos associados ao *C. hominis* nos EUA e, em um estudo realizado no Peru, foi considerado o subtipo mais virulento^5^. No Brasil, apenas um estudo investigou a presença do subtipo IbA10G2 e encontrou positividade em pacientes da mesma região do presente estudo^19^. A determinação dos subtipos auxilia na maior compreensão de como o parasito se comporta no hospedeiro, o que é importante para o desenvolvimento de novas estratégias para a prevenção e o tratamento da criptosporidiose^39^.

Do nosso ponto de vista, a inclusão de diagnósticos moleculares na rotina laboratorial é uma alternativa atraente ao diagnóstico microscópico, economizando de forma eficiente o tempo do laboratório. Além disso, a técnica não requer fluidos conservantes, como a formalina, que pode ser carcinogênica. É uma técnica com ótima sensibilidade e especificidade, que evita resultados falso-negativos e permite a diferenciação de parasitos descritos nos laudos como “complexos”, a exemplo de *E histolytica/E. dispar* ^40^.

O estudo apresenta limitações. A amostra é relativamente pequena e o grupo controle, que dependia de participação voluntária, foi menor do que o esperado. A decisão de incluir controles do mesmo domicílio que os pacientes em HD e o uso de técnicas avançadas de biologia molecular são os pontos fortes do estudo.

Nossos resultados indicam que os pacientes em HD não apresentaram taxas de prevalência mais elevadas de infecção por enteroparasitos. Espécies de protozoários foram encontradas com frequência nos testes coproparasitológicos de participantes provenientes de ambos os centros e em pacientes e controles, o que talvez reforce a necessidade de diagnóstico e tratamento de pacientes assintomáticos. Vale ressaltar que a detecção de *S. stercoralis* e *Cryptosporidium* spp. foi restrita a pacientes em HD. Nossos dados destacam a necessidade de implementação de técnicas diagnósticas não convencionais que possam atuar como um fator diferencial na detecção de enteroparasitos, especialmente em grupos específicos, como pacientes em HD.
